# Pathophysiological significance of cholesterol in ciliopathies

**DOI:** 10.20407/fmj.2025-044

**Published:** 2026-02-28

**Authors:** Takeshi Itabashi, Tomoka Morita, Moe Hirosawa, Daigo Kobayashi, Alamgir Hasan, Sotai Kimura, Hiroshi Itoh, Tatsuo Miyamoto

**Affiliations:** 1 Department of Molecular and Cellular Physiology, Graduate School of Medicine, Yamaguchi University, Ube, Yamaguchi, Japan; 2 Division of Advanced Genome Editing Therapy Research, Research Institute for Cell Design Medical Science, Yamaguchi University, Ube, Yamaguchi, Japan; 3 Department of Molecular Pathology, Graduate School of Medicine, Yamaguchi University, Ube, Yamaguchi, Japan

**Keywords:** Ciliopathy, Primary cilia, Peroxisome, Cholesterol, Autosomal dominant polycystic kidney disease

## Abstract

Primary cilia are sensory organelles essential for cellular signaling, the dysfunction of which causes ciliopathies, characterized by polycystic kidney disease, retinopathy, and developmental anomalies. Here, we review recent studies which have shown that cholesterol is a critical mediator of ciliary function and the pathogenesis of ciliopathies. Ciliary membranes show distinct cholesterol enrichment that is essential for the physiological function of various G-protein-coupled receptors and ion channels. Defects in *de novo* cholesterol synthesis (Smith–Lemli–Opitz syndrome) and peroxisome-facilitated cholesterol trafficking to ciliary membranes (Zellweger syndrome) result in ciliopathy-like phenotypes, establishing ciliary cholesterol insufficiency as a novel pathological mechanism. The polycystin complex associated autosomal dominant polycystic kidney disease localizes into ciliary membranes in a cholesterol-dependent manner. Specific cholesterol-binding sites in polycystin-2 are crucial for the distribution of cholesterol to cilia, while pathogenic mutations at these sites disrupt these interactions. We also discuss the previously underappreciated connections between ciliopathies, cholesterol, and other disorders such as cancer and neurodegenerative diseases, and opportunities for manipulating cholesterol for novel therapeutic strategies.

## Introduction

**Figure 1:**
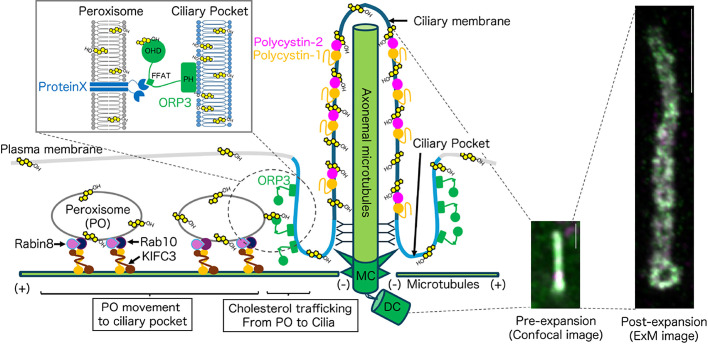
Model of ciliary cholesterol trafficking Peroxisomes move along microtubules and approach the ciliary pocket. Rabin8/Rab10/KIFC3 forms a molecular complex to enable peroxisome movement toward the ciliary pocket. Cholesterol is transported from the peroxisomal membrane to the ciliary membrane by ORP3. Cholesterol accumulates in the ciliary membrane. Confocal laser scanning microscopy and ultrastructural images of cholesterol (GRAM-W: accessible cholesterol probe, green) and polycystin-2 (red) within primary cilia (ARL13B, white) and centrosomes (FGFR1OP, white) of human retinal pigment epithelial (hTERT-RPE1) cells. Scale bar: 1 μm.

Primary cilia are nonmotile, microtubule-based, hair-like protrusive organelles originating from basal bodies (centrosomes) that develop on the cell surface during the quiescent G_0_ phase. The plasma membrane surrounding the microtubule array of the axoneme of primary cilia is known as the ciliary membrane. Enriched with a variety of ion channels and G-protein-coupled receptors, primary cilia are sensory organelles that detect signals from the extracellular environment. These signals include chemical cues and mechanical stimuli for development, tissue homeostasis, and cellular signaling pathways, including Hedgehog, Wnt, and PDGF signaling.^1^ Germline mutations of primary cilia-related genes cause ciliopathies, which affect an estimated 0.1% of individuals worldwide. These conditions are associated with a broad but specific spectrum of clinical features, including polycystic kidney disease, retinal degeneration, obesity, and developmental anomalies.^2,3^ To ensure the sensitive perception of extracellular stimuli, the membrane of primary cilia exhibits unique biochemical properties that distinguish it from other cellular membranes.^4^ Notably, this membrane contains higher concentrations of cholesterol (Figure 1), sphingolipids than other membranes, and specific proteins that facilitate its specialized sensory functions. The enrichment of cholesterol in ciliary membranes was first identified decades ago,^5–7^ but its functional significance remained poorly understood until recent breakthroughs revealed the critical role of cholesterol in maintaining ciliary integrity and signaling capacity.^8–14^

In this review, we summarize the multifaceted relationship between cholesterol and ciliopathies, concentrating on three areas: *de novo* cholesterol synthesis, the intracellular transport of cholesterol to cilia, and the functional significance of cholesterol in ciliary membranes. We also discuss the previously underappreciated connections between ciliopathies and neurodegeneration as they relate to cholesterol, highlighting potential shared pathogenic mechanisms and therapeutic targets.

## Cholesterol biosynthesis and ciliopathies

Cholesterol biosynthesis is a complicated, highly regulated process involving >30 enzymatic steps in the endoplasmic reticulum (ER) that convert acetyl-CoA to cholesterol through the mevalonate pathway. The initial and rate-limiting step is catalyzed by 3-hydroxy-3-methylglutaryl-coenzyme A (HMG-CoA) reductase, which produces mevalonate from HMG-CoA. Subsequent reactions involve the formation of isoprene units, their condensation to form squalene, cyclization to lanosterol, and finally conversion to cholesterol through a series of oxidation and reduction reactions.^15^ In the final step of cholesterol biosynthesis, 7-dehydrocholesterol is converted to cholesterol by 7-dehydrocholesterol reductase (DHCR7) and NADPH. Germline mutations in the *DHCR7* gene cause Smith–Lemli–Opitz syndrome (SLOS), which is characterized by gonadal dysgenesis and intellectual disability (Table 1).^16^ The clinical severity of SLOS has been suggested to correlate with the (decreased) level of blood cholesterol.^17^ Patients with severe SLOS and SLOS model mice develop symptoms typical of ciliopathies such as polycystic kidney disease, polydactyly, and other skeletal anomalies.^18,19^ It has also been reported that the inhibition of cholesterol synthesis through the application of HMG-CoA reductase inhibitors (statins) in zebrafish embryos can lead to the ciliopathy-related phenotype situs inversus.^20^ These findings suggest that an insufficient level of cellular cholesterol is a causative factor underlying the ciliopathy spectrum.

It was reported that statins significantly shorten the length of primary cilia;^20^ however, we observed no morphological abnormalities in primary cilia of skin fibroblasts derived from patients with SLOS or statin-treated healthy human-derived fibroblasts. Conversely, staining for cellular cholesterol using the probe filipin III revealed markedly decreased ciliary membrane cholesterol in cells from patients with SLOS compared with healthy cells.^10^ It has also been demonstrated that the impaired cilia-mediated Sonic hedgehog (Shh) signaling in cells from patients with SLOS can be restored by methyl-β-cyclodextrin (MβCD)-cholesterol supplementation.^10,21^ Patched1, the receptor for the Shh ligand, localizes to primary cilia in the absence of the ligand and regulates the amount of accessible cholesterol (free cholesterol not bound to sphingomyelin) in the outer ciliary membrane leaflet.^12,22,23^ When the Shh ligand binds to Patched1, the latter detaches from the cilia and instead the seven-transmembrane protein Smoothened is recruited to the ciliary membrane. Smoothened possesses a cysteine-rich domain (CRD) in its extracellular region. It is thought that the activation of Smoothened occurs via its incorporation of accessible cholesterol from the ciliary membrane; specifically, the binding of cholesterol to its CRD causes a conformational change in Smoothened.^24–27^ Smoothened has been reported to be activated not only by cholesterol but also by direct binding to oxysterols.^8,28^ Following the activation of Smoothened within cilia, Gli transcription factor processing occurs in the ciliary cytoplasm. The activated Gli transcription factor then translocates to the nucleus, where it induces the expression of Shh signaling response genes.^29^ When cells from patients with SLOS are treated with the Shh ligand, the accumulation of Smoothened in cilia is inhibited, thereby blocking proper Shh signaling.^10^ Because Shh signaling is an essential developmental program for digit patterning, these findings suggest it represents one mechanism by which polydactyly develops in SLOS.

## Ciliary cholesterol trafficking and ciliopathies

The cholesterol content in the ER, the primary site of cholesterol biosynthesis, accounts for only about 0.5%–1% of total intracellular cholesterol; the plasma membrane is highly enriched with cholesterol, containing approximately 50%–80% of the cellular content. This uneven distribution of cholesterol within the cell is due to its strict intracellular transport. In cells, a variety of lipids, including cholesterol, are transported via mechanisms involving vesicles and/or sites of contact between the membranes of different organelles.^30^ Among these, the best understood is the intracellular transport of low-density-lipoprotein (LDL)-derived cholesterol. LDL cholesterol binds to LDL receptors on the cell membrane and is internalized into the cell via clathrin-dependent endocytosis. It then passes through early endosomes, late endosomes, and lysosomes, before being transported to various organelles. In this process, esterified cholesterol within LDL cholesterol is converted to free cholesterol in endosomes/lysosomes. Niemann–Pick C2 protein (NPC2) in endosomes/lysosomes binds to free cholesterol and transfers it to Niemann–Pick C1 protein (NPC1), a lysosomal membrane protein. Defects in the *NPC1* and *NPC2* genes are causative of Niemann–Pick disease type C, an autosomal recessive genetic disorder characterized by hepatosplenomegaly and neurodegeneration (Table 1).^31^ In cells from patients with NPC, free cholesterol accumulates in endosomes/lysosomes, leading to decreased cholesterol levels in the plasma membrane. However, cholesterol levels in the ciliary membrane of cells from patients with NPC and *NPC1*-knockout cells are the same as in normal cells,^10^ which is consistent with the clinical observation that most patients with NPC do not develop typical ciliopathy symptoms such as polycystic kidney disease. In addition, while it was reported that primary cilia in the hippocampal region of *NPC1*-deficient mice become shorter and less responsive to Shh signaling, the associations of ciliary cholesterol levels with these changes have not been examined.^32^ Further research is needed to clarify whether NPC1 exerts differential effects on the formation and signaling of primary cilia depending on the organ. Nonetheless, findings to date suggest the existence of an NPC1/NPC2-independent transport pathway that supplies cholesterol to primary cilia.

Here, we focus on peroxisomes as the cellular component by which cholesterol is transported intracellularly to primary cilia (Figure 1). Peroxisomes are membrane-bound organelles responsible for lipid metabolism, including fatty acid β-oxidation and phospholipid synthesis.^33^ Zellweger syndrome (ZS) is an autosomal recessive disorder caused by mutations in peroxisome biogenesis genes (*PEXs*) (Table 1).^34^ Notably, ZS is associated with ciliary diseases such as polycystic kidney disease and retinitis pigmentosa, in addition to hepatosplenomegaly and hypotonia caused by lipid metabolism abnormalities. Deficiency of the peroxisomal bifunctional enzyme DBP/HSD17B4, which is clinically similar to ZS, causes dysregulation of primary cilia.^35^ These findings imply crosstalk between peroxisomes and primary cilia. Furthermore, screening for molecules involved in intracellular cholesterol transport using small interfering RNA libraries identified numerous peroxisomal molecules.^36^ Indeed, cells from patients with ZS both accumulate free cholesterol intracellularly and have less cholesterol in their ciliary membranes, which inhibits the localization of Smoothened in cilia.^10^ Cholesterol is required for the conversion of the Shh precursor protein into its active, mature, signaling form, but no abnormalities in such maturation were observed in cells from patients with ZS. Interestingly, it was reported that cells with knockout of the peroxisomal protein TMEM135 exhibit impaired ciliogenesis mediated by Rab8 inactivation, but the formation of primary cilia can be restored by cholesterol supplementation.^37^ ZS, like SLOS, is considered a ciliary disorder caused by cholesterol deficiency.

When viewed under an optical microscope, as many as hundreds of peroxisomes can be observed in a single cultured human cell. Live-cell imaging has revealed that some peroxisomes approach a region called the ciliary pocket, an invagination of the cell membrane at the base of primary cilia, and remain there for approximately 20 min.^10^ When cells are treated with inhibitors of microtubule polymerization, such movement of peroxisomes toward primary cilia is inhibited, and the ciliary cholesterol level decreases, suggesting that peroxisomes supply cholesterol to primary cilia.^10^ Furthermore, three-dimensional correlative light and electron microscopy/focused ion beam scanning electron microscopy revealed the formation of a membrane contact site between the peroxisome and the ciliary pocket.^10^ To identify molecules responsible for the peroxisome-mediated supply of cholesterol to cilia, using genome editing technology, we generated a cell library with the knockout of 180 candidate genes.^38,39^ We then screened for mutants exhibiting both decreased cholesterol levels in ciliary membranes and impaired peroxisome movement toward primary cilia. As a result, we discovered that the small G-protein Rab10 and its GTP exchange factor Rabin8, along with the kinesin molecule KIFC3 that moves toward the minus end of microtubules, form a complex on the peroxisomal membrane and function to bring peroxisomes and primary cilia close together (Figure 1).^10^ Furthermore, ORP3, one of the oxysterol-binding protein-related proteins, which act as intracellular cholesterol transporters,^40^ was shown to localize to the ciliary pocket and possess activity in transferring cholesterol from the peroxisomal membrane to primary cilia (Figure 1).^10^ In addition to the peroxisome-mediated transport mechanism, alternative pathways for transport of cholesterol into ciliary membranes have been proposed, including lysosome–peroxisome contacts,^36^ which regulate the mobile sterol pool accessible to peroxisomes, and *de novo* sterol synthesis near the ciliary base.^11^ The integration of these distinct mechanisms—local synthesis providing an immediate source and interorganelle contacts modulating the overall supply—likely enables highly regulated, tissue-specific maintenance of ciliary membrane homeostasis. Overall, we suggest that peroxisomes possess a key function of supplying cholesterol to primary cilia.

## Functions of cholesterol in cilia

Elucidating the mechanisms by which ciliary membrane cholesterol interacts with ciliary membrane proteins to exert physiological functions is an important challenge in cell biology. In pursuit of this goal, we focus on polycystic kidney disease as a clinical symptom commonly observed in SLOS and ZS, diseases associated with cholesterol insufficiency in the ciliary membrane. In physiological conditions, primary cilia on tubular and collecting duct epithelial cells sense urine flow and allow the entry of extracellular calcium ions to the cytoplasm, thereby negatively regulating the cAMP/protein kinase A (PKA) pathway to form the normal renal epithelial tubules.^41^ In polycystic kidney disease, hyperactivity of the cAMP/PKA pathway leads to increased cell proliferation and the secretion of substances such as chloride ions into the tubular lumen, raising the osmotic pressure (i.e., drawing water into the lumen). Consequently, the epithelial tubule expands and then cysts separate from the renal epithelial tubule structure. Such cysts continue to expand, increasing the total kidney volume and ultimately leading to renal failure.^41^ The calcium ion gate in cilia is a mechanosensitive ion channel complex called the polycystin complex.^42,43^ It is formed on the ciliary membrane by polycystin-1, which acts as a mechanosensor, and polycystin-2, which belongs to the transient receptor potential (TRP) ion channel family, at a 1:3 ratio.^44–47^ Germline mutations in the *PKD1* gene (encoding polycystin-1) and the *PKD2* gene (encoding polycystin-2) are causative of autosomal dominant polycystic kidney disease (ADPKD) (Table 1).^48^ The concept of the ciliary polycystin complex acting as a mechano-responsive Ca^2+^ channel has been challenged.^43^ However, mechanical stimulation responses involving influx through the cilia have been reported, potentially mediated by the release of calcium from the ER via the polycystin complex,^49^ or by other ciliary-localized TRP channels such as TRPV4.^50^ Thus, mechanosensing within the primary cilia is considered highly complex.

ADPKD is characterized by the enlargement of renal tubules and the formation of isolated, fluid-filled cysts in the kidneys and other organs such as the liver and pancreas. The majority of cases of ADPKD (74%) are attributed to mutations in the *PKD1* gene, while *PKD2* mutations account for approximately 24% of cases; the remaining cases arise from several genes including *GANAB*, *DNAJB11*, and *ALG8*.^51^ In about half of individuals with a heterozygous *PKD1* or *PKD2* mutation, ADPKD progresses slowly to end-stage renal disease.^48^ It is theorized that multiple cysts in patients develop through a cellular recessive “two-hit” mechanism involving both germline and somatic mutations in the *PKD1* or *PKD2* gene.^52,53^ An analysis integrating cryo-electron microscopy and molecular dynamics simulations identified a cholesterol-binding site on the surface of the six-transmembrane protein polycystin-2 that faces the outer leaflet.^54^ The steroid nucleus of cholesterol is accommodated within a hydrophobic pocket formed by a cluster of leucine (Leu517, Leu656), isoleucine (Ile561, Ile659), and valine residues (Val564, Val655).^54^ This pocket is situated between the S3 and S4 helices of the voltage sensor-like domain and the S6 helix of the pore domain. Of particular interest, a missense variant, L517R, which affects a residue in the cholesterol-binding site, is listed in the ADPKD genetic database (http://pkdb.pkdcure.org), where it is classified as “likely pathogenic.” Indeed, a pull-down assay confirmed that wild-type polycystin-2 bound cholesterol, but the polycystin-2 L517R mutant did not.^14^ We also used CRISPR/Cas9 technology to generate mice with the homozygous *PKD2* L517R mutation. These mice showed embryonic lethality, but the embryos exhibited typical ciliopathy phenotypes including situs inversus and kidneys with enlarged tubules and cysts. This confirmed that the failure of polycystin-2 to bind cholesterol causes a severe ciliopathy, leading to polycystic kidney disease *in vivo*.^14^ The findings suggest that ciliary cholesterol binding to polycystin-2 may be essential for proper functioning of the polycystin complex.

To clarify the pathophysiological significance of ciliary cholesterol in polycystic kidney disease, we investigated the channel activity and localization of the polycystin-2 L517R mutant protein. We first confirmed that this mutation did not impair the ion channel activity of polycystin-2, suggesting that its primary effect is on localization rather than channel function.^14^ However, it is possible that the ion channel activity of the polycystin-2 L517R protein was attributable to other sterol binding sites in polycystin-2. Indeed, it was reported that an oxysterol, 7β,27-dehydrocholesterol (7β,27-DHC), binds to the distinct pocket site formed by the pre-S1 helix and the S4 and S5 linker of polycystin-2 and controls ciliary ion channel activity *in vitro*.^13^ It was also reported that ciliary localization of polycystin-2 mutant proteins with defective 7β,27-DHC binding was not impaired.^13^ However, because the data were obtained from cells overexpressing those mutants, they may not reflect precise localization, and the possibility remains that the 7β,27-DHC binding site also controls ciliary polycystin-2 localization. To overcome such concerns about overexpression experiments in ciliated cultured cells, we used a CRISPR/Cas9 technology-mediated variant knock-in approach to evaluate ciliary localization of the polycystin complex. In a cell line derived from mouse kidney collecting tube (mIMCD-3) cells with the L517R mutation of *Pkd2*, the amount of polycystin-2 in primary cilia was drastically decreased compared with that in wild-type cells. Interestingly, we demonstrated that the ciliary cholesterol level was also significantly decreased in *Pkd2* L517R mutant cells and *Pkd2*-knockout cells, and that intracellular cholesterol aggregated outside primary cilia in both of these cell types. Polycystin-2 is known to localize not only to primary cilia but also to the ER,^49^ suggesting that defective intracellular cholesterol supply to the cilia in the *Pkd2* mutant cells decreases the level of ciliary cholesterol. We next confirmed that extracellular supply of cholesterol to *Pkd2* L517R mutant cells could not restore the ciliary localization of the PKD2 protein. In contrast to the L517R mutant, which showed impaired ciliary localization and decreased cholesterol levels, the impaired ciliary localization W414G mutant retained its cholesterol-binding ability, and ion channel activity^14,55^ was restored upon cholesterol supplementation. We also found that decreasing ciliary cholesterol levels, either through chemical treatment (MβCD) or by deleting the *Pex14* gene encoding a peroxisomal protein (a ZS model), significantly decreased the levels of polycystin-1 and polycystin-2 in primary cilia.^14^ Conversely, adding water-soluble cholesterol restored the localization of both polycystins to normal levels. Furthermore, in 3D-mIMCD-3 cell culture, both MβCD treatment and *Pex14* deletion caused enlargement of the lumen in cell spheroids, a hallmark of polycystic kidney disease. Notably, adding external cholesterol to the *Pex14*-knockout cells rescued the lumen size, restoring it to wild-type levels. These findings suggest that the ability of polycystin-2 to directly bind cholesterol supplied to cilia by peroxisomes is essential for the stable accumulation of cholesterol in primary cilia. It is deficiency in this accumulation of cholesterol, rather than in ion channel regulation, by which polycystin mutations result in polycystic kidney disease. As such, interventions targeting polycystin-2 rather than ion channel regulation may be more effective for preventing polycystic kidney disease.

## Future perspectives

### How does cholesterol control the ciliary localization of polycystin?

Live-cell imaging of Pkd2–green fluorescent protein (GFP) expressed in *Pkd2*-knockout mIMCD-3 cells showed that MβCD treatment gradually decreased the ciliary signal of polycystin-2–GFP fusion protein near the basal body, indicating that pre-existing ciliary polycystin-2 proteins are internalized through lateral diffusion.^14^ Our findings suggest that ciliary cholesterol captures polycystin-2 in the primary cilia compartment and that the cholesterol–polycystin complex that forms has decreased mobility, leading to accumulation of the complex in the ciliary membrane.^14^ Further comparison of the conformations between wild-type polycystin-2 and the polycystin-2 L517R mutant protein should reveal the mechanism of cholesterol-mediated ciliary accumulation of the polycystin complex.

### Potential drug therapies for ADPKD

The vasopressin V2 receptor antagonist tolvaptan is approved globally as a treatment for ADPKD, but caution is required when considering this treatment because of its potent diuretic effect, which can lead to dehydration and hyponatremia.^56^ Furthermore, this drug has only been prescribed since the designation of ADPKD as an intractable disease, and its efficacy and safety have not been confirmed for patients already undergoing dialysis, pregnant women, or children. The vitamin A receptor agonist tamibarotene shows suppressive effects against polycystic kidney in mice and human kidney organoids,^57^ and is currently undergoing phase II clinical trials. However, this agent is known to be teratogenic.^58^ Furthermore, development is underway for RGLS4326 (anti-miR-17), which targets miR-17, a microRNA highly expressed in patients with ADPKD.^59^ Generally, miRNA therapeutics face challenges such as hepatotoxicity, inadequate drug delivery systems, and the need for repeated injections. Consequently, unmet medical needs persist for ADPKD. Against this background, ciliary cholesterol is expected to serve as a target for drug discovery for ADPKD, because it localizes the polycystin complex to the primary cilia, which in turn prevents the development of polycystic kidney disease.

### Targeting ciliary cholesterol as a therapeutic target: Beyond ciliopathies

Primary cilia generally disappear when quiescent G_0_ phase cells receive the proliferative signals to re-enter the cell cycle.^60–63^ Primary cilia can thus not be identified in many malignant and proliferative tumors such as pancreatic, small intestinal, and colorectal cancers.^64,65^ However, various cancers, including basal cell carcinoma, medulloblastoma, gastrointestinal stromal tumor, claudin-low breast cancer, bladder cancer, and others, retain their primary cilia, which sense oncogenic Shh and Wnt, which are involved in tumorigenesis and tumor progression.^64,65^ It was reported that statins decrease the recurrence and mortality of cancer by prohibiting the outgrowth of dormant cancer cells in various tissues.^66–69^ Contrasting therapeutic strategies reflect the dual roles of cholesterol in cancer: Statins are primarily considered for dormant/quiescent tumors (lacking primary cilia) by generally restricting the bulk availability of lipids for cell proliferation in the cytoplasm; conversely, ciliary cholesterol agents could be used to modulate or inhibit oncogenic ciliary signaling (e.g., Shh, Wnt) in tumors that retain their primary cilia, by selectively lowering ciliary cholesterol. Clarification is crucial to define the specific patient populations and signaling pathways relevant to each approach. To apply statins and ciliary cholesterol agents as cancer therapeutics, there is also a need for further investigations, to clarify which primary cilia-related oncogenic signals are involved in the ciliary accumulation of cholesterol.

Recent 3D-electromicroscopy observations in the brain revealed that excitatory and inhibitory neurons, astrocytes, and oligodendrocyte progenitor cells have primary cilia, while microglia and mature oligodendrocytes do not.^70–73^ Primary cilia in the brain are rich in receptors for various neurotransmitters and neuropeptides, including for serotonin, dopamine, and somatostatin. This allows them to modulate neuronal excitability and circuit formation.^74^ While primary cilia and synaptic junctions are physically distinct, they are functionally linked.^74^ For example, primary cilia are often located near the axon initial segment, a critical site for action potential generation. There, they are thought to regulate synaptic plasticity and signaling intensity. Recent advances have begun to elucidate the pathophysiological significance of primary cilia in neurodegenerative diseases such as Parkinson’s disease (PD) and spinocerebellar ataxia (SCA).^75–78^ Notably, mutations in *GBA1* (encoding β-glucoceramidase), which cause Gaucher disease and are the most common genetic cause of familial PD, impair primary cilia-related Shh signaling and decrease the levels of neuroprotective glial cell line-derived neutrophic factor and neurturin, which maintain the survival and function of dopamine neurons in the dorsal striatum.^79^ Interestingly, it was reported that cells from patients with *GBA1*-related PD showed a decrease in the level of ciliary cholesterol, and that cholesterol supplementation to the cells restored primary cilia-related Shh signaling.^79^ Furthermore, it was demonstrated that mutation in *TTBK2*, which causes SCA11, significantly impairs the transport of peroxisomes to primary cilia, leading to decreased Shh signaling.^80^ Cholesterol supplementation restored the ciliary cholesterol-related phenotype in cells from patients with SCA11, suggesting that ciliary cholesterol might be a promising new therapeutic target for both PD and SCA.^80^ Considering that primary cilia-related genes including *NEK1*^81^ and *CFAP410/C21ORF2*^82,83^ have been reported as causative genes of amyotrophic lateral sclerosis (ALS), that genes causative of ALS are involved in ciliogenesis,^84^ and that a correlation between cholesterol metabolism abnormalities and ALS onset has been identified,^85^ it is important to elucidate the pathophysiological significance of ciliary cholesterol broadly for neurodegenerative diseases.

The role of cholesterol in cilia has opened new avenues for therapeutic intervention. Potential translational strategies include cyclodextrin-based cholesterol delivery to rescue ciliary membrane integrity, administration of statins to target cholesterol synthesis in certain cancers or neurodegenerative diseases, and using small-molecule Smoothened modulators, whose activity is closely linked to ciliary cholesterol levels. These approaches offer possibilities for compensating for loss-of-function mutations and regulating cholesterol-sensitive ciliary signaling. A line of recent studies has suggested that ciliary cholesterol, as revealed by staining using expansion microscopy technology (ExM) and the GRAM-W probe for accessible cholesterol^86^ (Figure 1), may serve as a therapeutic target not only for ciliopathies but also for a broader range of diseases. Two major challenges lie ahead for drug development targeting ciliary membrane cholesterol. First, it is essential to evaluate the precise threshold (quantity, accessibility, and localization) of ciliary membrane cholesterol deficiency that triggers ciliopathic phenotypes. Furthermore, the optimal level of ciliary membrane cholesterol required to suppress ciliopathy might vary significantly in different tissues. Second, given that cholesterol is ubiquitously present and performs critical functions outside the cilium, such as lipid raft formation, any potential systemic side effects associated with ciliary membrane cholesterol manipulation must be assessed rigorously. Addressing these critical challenges is expected to accelerate both fundamental and translational research focused on ciliary membrane cholesterol.

## Figures and Tables

**Table 1:** Intracellular cholesterol transport-related disorders and associated ciliary cholesterol levels

Disease	Associated genes	Typical ciliopathy-like symptoms	Intracellular cholesterol accumulation	Ciliary cholesterol level
Smith–Lemli–Opitz syndrome	*DHCR7*	polycystic kidney, polydactyly	–	low
Zellweger syndrome	*PEX1*, *PEX2*, *PEX3*, *PEX5*, *PEX6*, *PEX10*, *PEX11B*, *PEX12*, *PEX13*, *PEX14*, *PEX16*, *PEX19*, *PEX26*	polycystic kidney, retinitis pigmentosa	+	low
X-linked adrenoleukodystrophy	*ABCD1*	N.D.	+	normal
Nieman–Pick disease type C	*NPC1*, *NPC2*	N.D.	+	normal
Autosomal dominant polycystic kidney	*PKD1*, *PKD2*	polycystic kidney, polycystic liver, polycystic pancreas	+	low

N.D.: not detected
